# The Impact of Local Tranexamic Acid Infiltration on Periorbital Ecchymosis and Edema in Rhinoplasty: A Triple-Blinded Randomized Controlled Trial

**DOI:** 10.1093/asjof/ojaf172

**Published:** 2025-12-23

**Authors:** Saeed Golparvaran, Ali Aalizade, Farhad Arbabzade, Benyamin Rahmaty, Farrokh Heidari, Behrouz Amirzargar, Amir Mahdi Mohamadi Kamal, Arshia Mobini, Nima Nazari, Mohammad Sajjad Parvin

## Abstract

**Background:**

Rhinoplasty is acknowledged as one of the most commonly conducted facial plastic surgical procedures worldwide. Periorbital ecchymosis and edema pose considerable postoperative complications that influence patient satisfaction and recovery duration.

**Objectives:**

This study aimed to examine the impact of local tranexamic acid (TXA) infiltration on periorbital ecchymosis and edema in patients undergoing rhinoplasty.

**Methods:**

A randomized, triple-blind, controlled trial was conducted involving 80 patients scheduled for primary open septorhinoplasty at our university hospital between 2021 and 2022. Participants were randomly allocated to receive either local infiltration consisting of TXA (1 mg/mL) combined with lidocaine and epinephrine (*n* = 40) or a control solution containing identical components, except TXA (*n* = 40). Periorbital ecchymosis and edema were evaluated at 24 h and 1 week postoperatively, utilizing a standardized scale.

**Results:**

No significant differences were observed between the TXA and control groups regarding periorbital edema at 24 h (*P* = .965) and 1 week (*P* = 1.000) postoperatively. Additionally, periorbital ecchymosis showed no substantial difference between the groups at 24 h (*P* = .597) or 1 week (*P* = .063). Intraoperative bleeding was marginally reduced in the TXA group (39.38 ± 23.699 vs 43.13 ± 26.435 mL), although this difference did not achieve statistical significance (*P* = .506).

**Conclusions:**

The local infiltration of TXA did not lead to a statistically significant reduction in periorbital edema, ecchymosis, or intraoperative bleeding among rhinoplasty patients compared with a placebo. These findings suggest that local infiltration may not provide the same benefits in rhinoplasty procedures as those documented with systemic administration routes.

**Level of Evidence: 2 (Therapeutic):**

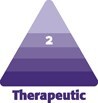

Rhinoplasty is a form of facial reconstructive surgery designed to enhance the aesthetic appearance and functionality of the nose.^1,2^ It remains among the most commonly performed facial plastic surgical procedures in Iran and globally.^3^ The prevalence rate in Iran is as high as 180 rhinoplasty cases per 100,000 individuals, representing the highest rate in the world.^4^ Despite advancements in surgical techniques, postoperative complications, including periorbital ecchymosis and edema, continue to pose significant concerns for both patients and surgeons. Osteotomy conducted during rhinoplasty may inadvertently injure local blood vessels, leading to undesirable results that can prolong recovery periods and affect overall patient satisfaction.^5^ Furthermore, rhinoplasty may result in considerable blood loss during the procedure, even when employing controlled hypotension during anesthesia, potentially necessitating blood transfusions in severe instances.^6^ Excessive hemorrhaging compromises the visibility of the surgical field and escalates the risk of postoperative complications.^7^

Various strategies have been employed to mitigate these complications, including lowering blood pressure during surgery, elevating the head, applying local cooling, using intranasal packing, administering epinephrine solutions, utilizing decongestants, fibrin sealants, and medications such as bromelain to reduce postrhinoplasty bruising.^8^ Additionally, ketamine and lidocaine are used to manage emergence agitation and postoperative pain.^9^ Tranexamic acid (TXA) is an antifibrinolytic agent used to reduce bleeding and postoperative edema and helps in controlling both intraoperative and postoperative issues.^10^ Among the pharmacological interventions, TXA has gained particular attention because of its antifibrinolytic properties, which effectively reduce surgical bleeding and the need for blood transfusions.^11^ TXA is an analog of lysine and binds to plasminogen and plasmin, inhibiting their ability to bind to lysine residues in fibrin and thus inhibiting fibrinolysis.^12^ It has been widely used to reduce intraoperative blood loss and postoperative edema and ecchymosis in aesthetic facial plastic surgery, primarily in rhinoplasty, rhytidectomy, and blepharoplasty, as well as in major surgeries, including open-heart surgery under extracorporeal circulation, coronary artery bypass surgery, and hip arthroplasty.^13-17^ Although generally considered safe, TXA in rare cases may cause adverse effects such as deep vein thrombosis and pulmonary embolism.^11^

Previous studies have examined the effectiveness of oral and intravenous TXA in rhinoplasty. Recent meta-analytical evidence has concluded that TXA reduces average blood loss by 41.6 mL while simultaneously diminishing periorbital ecchymosis and edema.^11^ Similarly, injectable TXA has proven effective in lessening postoperative ecchymosis and edema in facelift surgeries.^18^ Recent research has further indicated that local infiltration of TXA during facelift procedures resulted in reduced bleeding, shorter operation times, and decreased postoperative drain secretions.^19^ However, the impact of local infiltration of tranexamic acid specifically on periorbital ecchymosis and edema following rhinoplasty remains insufficiently explored in the literature.

In light of this knowledge deficiency, we conducted a randomized, triple-blind study involving 80 patients to examine the impact of local TXA infiltration on postoperative ecchymosis and periorbital edema in individuals undergoing rhinoplasty. Our research aims to assess whether this methodology effectively mitigates common complications associated with rhinoplasty, thereby contributing to the ongoing effort to develop enhanced perioperative management strategies.

## METHODS

This study was conducted as a randomized, triple-blind, controlled trial to assess the effect of local TXA on periorbital ecchymosis and edema in patients undergoing septorhinoplasty surgery at our university hospital from 2021 to 2022. In this triple-blind, randomized control trial, 80 candidates for rhinoplasty were equally and randomly assigned to 2 groups: one receiving TXA (*n* = 40) and the other serving as a control (*n* = 40). The study population consisted of patients referred to the hospital who were candidates for primary open septorhinoplasty surgery. Utilizing a priori power analysis (effect size *d* = 0.81, *α* = .05, power = 0.80, allocation ratio = 1), a total sample size of 80 patients, with 40 patients allocated to each group, was deemed necessary.

### Inclusion and Exclusion Criteria

Patients were eligible for inclusion if they were between 18 and 45 years old, had no documented medical history, no documented smoking history, had complete medical records, were undergoing primary rhinoplasty, and did not require rib or ear cartilage grafting. Additionally, patients had to be scheduled for septorhinoplasty without any concurrent procedures such as blepharoplasty or facelift.

The criteria for exclusion comprised individuals who are younger than 18 years or older than 45 years, cases involving revision or retouch rhinoplasty, the necessity for ear or rib cartilage, the existence of congenital disorders (eg, cleft lip), candidates for combined procedures beyond rhinoplasty, severe septal deviation, or marked bony vault deformity, the presence of concha bullosa, and contraindications to TXA (which encompass drug sensitivity, a history of coagulation disorders, deep vein thrombosis, color vision disorders, or cerebral hemorrhage). Patients with blood pressure exceeding 130 mm Hg systolic and 80 mm Hg systolic during the procedure were excluded. Patients were excluded if intraoperative deviation from the standardized surgical protocol occurred (eg, alternative osteotomy approach or additional grafting).

### Ethical Considerations

All participants provided written informed consent following a comprehensive explanation of the procedure, which included its benefits and potential risks. The study protocol was approved by the Ethics Committee of Tehran University of Medical Sciences (Ethical Committee Registration Number: IR.TUMS.MEDICINE.REC.1400.1182).

### Randomization and Blinding

Block randomization was employed to ensure equilibrium among the groups. A total of 10 blocks, each containing 8 participants, were established. Using a random number table, 4 participants in each block were randomly allocated to either the intervention or control group. Consequently, this methodology resulted in 40 participants being assigned to each group. A triple-blind approach was implemented: the otolaryngologist performing the injections, the patients and the analyst (different from the primary surgeon) were all blinded to group allocation. An operating room nurse prepared all syringes, which appeared identical in shape and color, and were identified only by numbers (1-40) corresponding to group allocation on a separate form inaccessible to the surgeon, patients, and analyst.

### Interventions

All patients underwent standardized anesthesia induction utilizing midazolam (0.02-0.04 mg/kg), fentanyl (2-4 μg/kg), propofol (1-2.5 mg/kg), and atracurium (0.5 mg/kg). Anesthesia was sustained with propofol (50-150 μg/kg/min) and remifentanil (0.1-0.4 μg/kg/min). Atracurium (0.1 mg/kg) was administered as required to preserve muscle relaxation, and fentanyl (50 μg) was administered for pain management when deemed necessary. A minimum systolic blood pressure of 80 mm Hg was also considered for all cases.

Local injection was performed on all patients following intubation and 15 min before surgery. The control group received the standard solution, which consisted of 5 mL of 5% lidocaine, 0.1 mL of 1:1000 epinephrine, and 4.9 mL of distilled water. Conversely, the intervention group received a modified solution formulated with 5 mL of 5% lidocaine, 0.1 mL of 1:1000 epinephrine, 1 mL of TXA solution (10 mg/mL), and 3.9 mL of distilled water, resulting in a final concentration of 1:100,000 epinephrine and 1 mg/mL of TXA (as per Kochuba's article).^19^ Distilled water was used as a diluent based on Kochuba's protocol for local TXA preparation in facelift surgery, as it ensured reproducibility of concentration across all patients and avoided potential confounding from electrolytes present in saline or Ringer's lactate.

The TXA solution was prepared by diluting 1 mL from a 500 mg/5 mL vial with distilled water in a 1:10 ratio to achieve a 10 mg/mL concentration. Local injections were administered at the base of the columella, the tip area, the bony hump, and bilaterally at the lateral osteotomy sites. All patients received prophylactic intravenous cefazolin (1 g) and dexamethasone (8 mg) at the commencement of the procedure.

### Surgical Procedure

All operations were performed by a single senior surgeon using a standardized open structural rhinoplasty protocol, minimizing variability in technique and intraoperative handling. The procedure commenced with a columellar skin incision, which was followed by the elevation of the skin and the creation of a mucoperichondrial flap. The cartilaginous and bony hump was excised, and standard septoplasty was conducted. Auto spreader flaps were prepared, and tip plasty was performed and secured utilizing the tongue-in-groove technique, subsequent to which lateral osteotomy was conducted employing the internal low-to-low method, and transverse osteotomy was executed using the external technique. For all cases, subperiosteal dissection before osteotomy was performed to minimize the soft tissue trauma. Following the osteotomy, the site was compressed with cold, normal saline-soaked gauze for 5 min. Ultimately, the skin was sutured, and alar base resection was conducted as necessary. This procedure was performed on all cases. If the surgery method were different, cases would have to be excluded from the study.

Postoperatively, all patients were prescribed acetaminophen 325 mg, administered orally as 2 tablets every 4 to 6 h as needed, for pain management during the initial 5 days following surgery.

All patients received standard postoperative care, including external and internal nasal splinting for 7 days. They were instructed to keep their heads elevated and apply cold compresses intermittently during the first 48 h. All cases were managed as outpatient procedures and discharged on the same day.

### Follow-up, Data Collection, and Measurement

Standardized photographs were captured 24 h and 1 week postoperatively for scoring.

**Figure 1.**
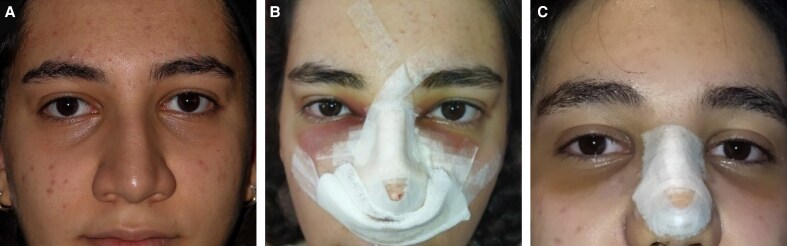
Tranexamic acid (TXA) group patient. Clinical photographs of a 23-year-old female patient in the TXA group. (A) Preoperative appearance. (B) Postoperative Day 1: periorbital edema Grade 2, ecchymosis Grade 3. (C) Postoperative Day 7: periorbital edema Grade 1, ecchymosis Grade 2.

**Figure 2.**
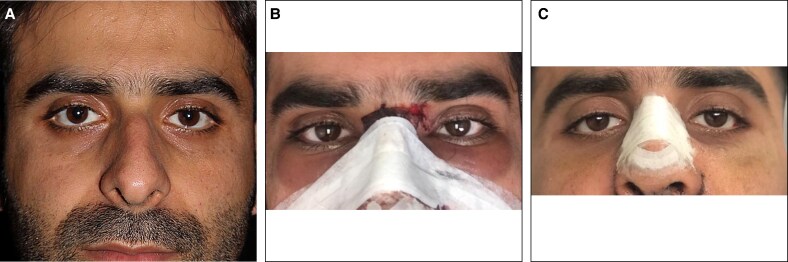
Control group patient. Clinical photographs of a 27-year-old male patient in the control group. (A) Preoperative appearance. (B) Postoperative Day 1: periorbital edema Grade 2, ecchymosis Grade 4. (C) Postoperative Day 7: periorbital edema Grade 1, ecchymosis Grade 1.

The primary outcome focused on the severity of periorbital edema and ecchymosis, which was assessed 24 h and 1 week postrhinoplasty. The secondary outcome measured the volume of intraoperative bleeding. All postoperative photographs were evaluated by an independent otolaryngologist with more than 5 years of surgical experience, who was not involved in the operations and was blinded to group allocation. Periorbital ecchymosis and edema were evaluated utilizing the scale delineated in Mehdizadeh's 2017 article.^10^ The full grading scales for periorbital edema and ecchymosis are provided in Supplemental Table 1. According to this scale, both ecchymosis and edema were assigned scores ranging from 0 to 4, reflecting their severity. Photographs were captured at 24 h and 1 week postoperatively (Figures 1, 2), and scoring was conducted based on these images. The scores for ecchymosis and edema at both time points were assessed for each group, and the percentage distribution of scores was compared across the groups. Blood loss was quantified using suction canister measurement (after accounting for irrigation) and gauze weighing.

As an ancillary outcome measure, we assessed intraoperative hemorrhage during rhinoplasty procedures. Intraoperative blood loss was measured by subtracting the volume of irrigation fluid from the total suction canister volume and adding the weight of blood-soaked gauze (1 g = 1 mL). Volumes were recorded in milliliters, and decimals reflected the digital scale readings rather than an absolute precision to the tenth of a milliliter.

### Statistical Analysis

Data were systematically entered into SPSS Statistics for Windows, Version 26.0 (IBM Corp., Armonk, NY) for thorough analysis and interpretation. Quantitative data were reported as means with standard deviations or medians with interquartile ranges, whereas qualitative data were presented as frequencies and percentages. To compare variables between groups after the intervention at both 24 h and 1 week postoperatively, independent *t*-tests or Mann–Whitney *U* tests were used depending on the results of normality testing. Paired *t*-tests or Wilcoxon signed-rank tests were employed for comparisons within each group. Chi-squared tests were used for categorical variables. A *P*-value of <.05 was considered statistically significant.

## RESULTS

The mean age of patients in the TXA group was 31.38 ± 8.161 years, compared with 29.93 ± 8.365 years in the control group. Concerning gender distribution, both groups exhibited a higher percentage of women, with 60% in the TXA group and 72.5% in the control group. No statistically significant differences were noted between the 2 groups in terms of mean age or gender distribution (*P* > .05), as illustrated in Table 1.

**Table 1.** Demographic Characteristics of Rhinoplasty Candidates

Variable	TXA group (*n* = 40)	Control group (*n* = 40)	*P*-value
Age (Mean ± SD)	31.38 ± 8.16	29.93 ± 8.37	.435
Gender: male	16 (40%)	11 (27.5%)	.237
Gender: female	24 (60%)	29 (72.5%)	

SD, standard deviation; TXA, tranexamic acid.

### Periorbital Edema

#### Edema at 24 h Postoperation

**Figure 3.**
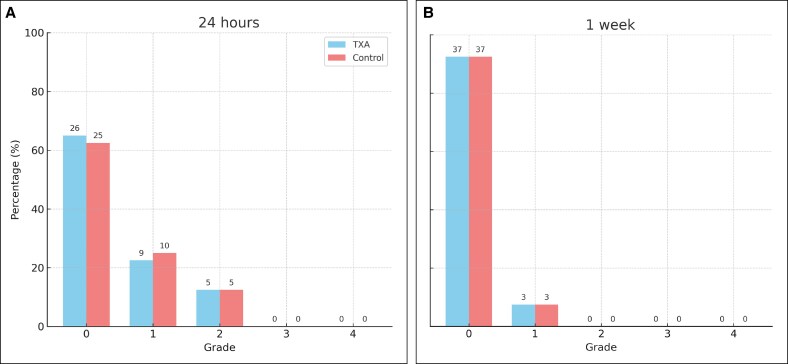
Periorbital edema after rhinoplasty in the tranexamic acid (TXA) and control groups. (A) Edema severity at 24 h postsurgery. (B) Edema severity at 1 week postsurgery. Data are expressed as percentages; numbers inside bars represent patient counts.

At 24 h following rhinoplasty, no significant difference was noted between the TXA and control groups (*P* = .965). Within the TXA group, 65% of patients (*n* = 26) exhibited no edema, 22.5% (*n* = 9) experienced Grade 1 edema (minimum coverage of the iris), and 12.5% (*n* = 5) presented with Grade 2 edema (relative iris coverage). In the control group, the distribution was similar, with 62.5% (*n* = 25), 25% (*n* = 10), and 12.5% (*n* = 5), respectively (Figure 3A). The full grading scales for periorbital edema and ecchymosis are provided in Supplemental Table 1.

#### Edema at 1 Week Postoperation

One week following rhinoplasty, both groups exhibited comparable outcomes with no statistically significant difference (*P* = 1.000). In the TXA and control groups, 92.5% of patients (*n* = 37) experienced the absence of edema, whereas 7.5% (*n* = 3) exhibited Grade 1 edema. No patients in either group presented with Grade 2 or higher edema (Figure 3B).

### Periorbital Ecchymosis

#### Ecchymosis at 24 h Postoperation

**Figure 4.**
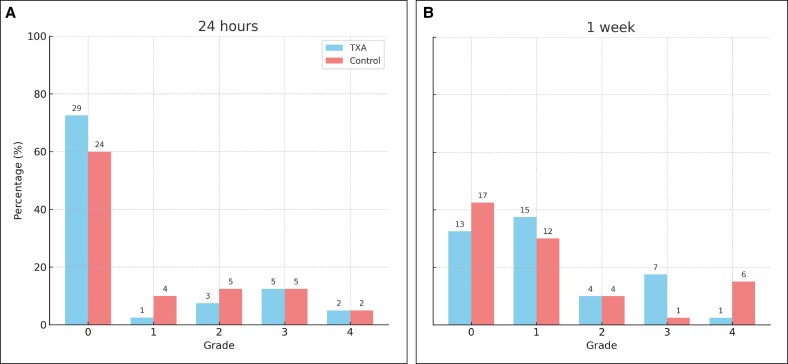
Periorbital ecchymosis after rhinoplasty in the tranexamic acid (TXA) and control groups. (A) Ecchymosis severity at 24 h postsurgery. (B) Ecchymosis severity at 1 week postsurgery. Data are expressed as percentages; numbers inside bars represent patient counts.

The severity of ecchymosis was assessed utilizing the Mehdizadeh scale.^10^ At 24 h postrhinoplasty, no significant differences were noted between the 2 groups (*P* = .597). In the TXA group, 72.5% of patients (*n* = 29) exhibited no ecchymosis, 2.5% (*n* = 1) presented with Grade 1, 7.5% (*n* = 3) displayed Grade 2, 12.5% (*n* = 5) showed Grade 3, and 5% (*n* = 2) experienced Grade 4 ecchymosis (which extended to the lateral canthus). In the control group, 60% of patients (*n* = 24) exhibited no ecchymosis, 10% (*n* = 4) were classified with Grade 1, 12.5% (*n* = 5) exhibited Grade 2, 12.5% (*n* = 5) had Grade 3, and 5% (*n* = 2) presented with Grade 4 ecchymosis (Figure 4A).

#### Ecchymosis at 1 Week Postoperation

At 1 week postrhinoplasty, no statistically significant difference was observed between the TXA and control groups (*P* = .063). Within the TXA group, 32.5% of patients (*n* = 13) exhibited no ecchymosis, 37.5% (*n* = 15) presented with Grade 1, 10% (*n* = 4) with Grade 2, 17.5% (*n* = 7) with Grade 3, and 2.5% (*n* = 1) with Grade 4 ecchymosis. Conversely, in the control group, 42.5% of patients (*n* = 17) demonstrated no ecchymosis, 30% (*n* = 12) exhibited Grade 1, 10% (*n* = 4) Grade 2, 2.5% (*n* = 1) Grade 3, and 15% (*n* = 6) Grade 4 ecchymosis (Figure 4B).

### Intraoperative Bleeding

**Figure 5.**
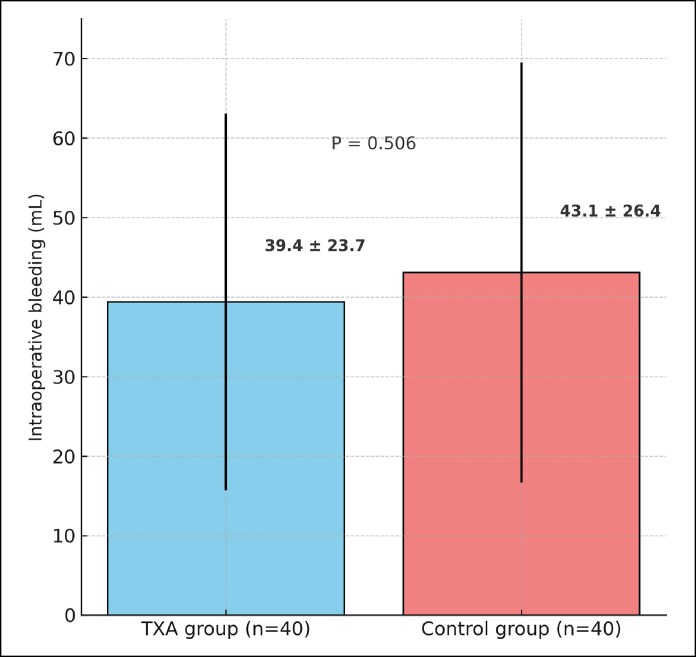
Comparison of intraoperative bleeding volume between groups. Mean ± standard deviation intraoperative blood loss is shown for the tranexamic acid (TXA) group (*n* = 40) and control group (*n* = 40). No statistically significant difference was observed (*P* = .506).

The mean blood loss was 39.38 ± 23.699 mL in the TXA group, whereas it was recorded at 43.13 ± 26.435 mL in the control group. According to the independent *t*-test, no significant difference was observed between the 2 groups (*P* = .506; Figure 5). Given the substantial standard deviation observed in the bleeding measurements, supplementary nonparametric testing was conducted. The Mann–Whitney test revealed no significant difference in intraoperative bleeding between the 2 groups (*P* = .840).

## DISCUSSION

This randomized, triple-blind investigation examined the impact of localized infiltration of TXA on periorbital edema, ecchymosis, and intraoperative bleeding in patients undergoing rhinoplasty. The results of our study demonstrate that the local administration of TXA did not significantly decrease these complications when compared with the placebo group.

Rhinoplasty surgery is one of the most prevalent procedures for addressing aesthetic concerns and respiratory issues. Postoperative complications, including periorbital edema and ecchymosis, can result in unsatisfactory experiences for patients, particularly because of the diminutive size of the nasal cavity and the abundance of blood vessels, which complicate the control of bleeding. Numerous techniques have been suggested to alleviate these challenges, and TXA has emerged as a promising adjunct through various administration routes.^20^ Recent systematic reviews of rhinoplasty trials have illustrated that preoperative systemic TXA, whether administered orally or intravenously, significantly diminishes intraoperative blood loss as well as periorbital edema and ecchymosis during the first week postoperatively, indicating that systemic TXA may yield significant advantages in surgical interventions.^1,21^

Numerous clinical trials have demonstrated significant benefits from administering systemic TXA. Beikaei et al reported notable reductions in intraoperative bleeding with intravenous TXA, whereas Sakallioğlu et al found that oral administration reduced postoperative edema and ecchymosis.^3,5^ Similarly, Eftekharian and Rajabzadeh demonstrated a decrease in surgical duration and an improvement in surgeon satisfaction with the use of oral TXA.^6^ Mehdizadeh et al determined that TXA resulted in a reduction of blood loss by an average of 41.6 cc, while also decreasing ecchymosis and periorbital edema.^10^ Sagiv et al conducted a randomized controlled trial (RCT) of the local injection of TXA along with lidocaine before making incisions in upper eyelid blepharoplasty. Upon examination during the first postoperative week, they reported no significant reduction in ecchymosis.^22^

Avci examined various dosages of TXA in relation to reducing blood loss during rhinoplasty procedures. The findings indicated that the administration of either a single dose or intermittent doses of intravenous TXA resulted in a decrease in total bleeding. However, intermittent dosing did not demonstrate superiority over administering a single dose.^21^

Of significant relevance to our approach, Kochuba et al's study demonstrated that local infiltration of TXA during facelift surgery led to a reduction in bleeding, operational time, and drain secretions.^19^ Couto et al established that TXA infiltration mitigated bleeding in facelift surgery.^23^ Additionally, Cohen et al reported that intravenous TXA administered during a facelift substantially diminished postoperative bruising and serous fluid collection.^18^ The conclusions drawn from these previous studies prompted us to explore whether analogous benefits from TXA could be realized in rhinoplasty through local infiltration rather than systemic administration.

Notwithstanding these encouraging precedents, our comparison involving 40 patients who received local TXA infiltration and 40 who were administered a placebo revealed no statistically significant differences in periorbital edema at 24 h or 1 week postoperation. Likewise, although ecchymosis appeared diminished in the TXA group, this difference failed to attain statistical significance at both intervals. The intraoperative bleeding volume was reduced in the treatment group, although it did not reach statistical significance. Baseline characteristics, including age (*P* = .435) and gender distribution (*P* = .237), were comparable between groups, confirming similarity at baseline rather than indicating an effect on outcomes, which is consistent with previous research on demographic factors and postoperative complications.

The present results diverge from those reported by Vaghardoost et al, who conducted a randomized trial of localized TXA injection (10 mg/kg) in rhinoplasty, noting a significant reduction in intraoperative hemorrhage, as well as postoperative periorbital edema and ecchymosis.^24^ The disparity observed between our findings and those of earlier studies is likely attributable to variations in administration routes, dosages, and drug distribution. This contrast likely reflects differences in dosage (10 mg/kg vs 1 mg/mL), administration techniques, and patient selection. Consistent with our investigation, the survey conducted by Afzali et al indicated that the reduction in blood loss during surgery in the TXA-administering group did not differ significantly from that observed in the control group.^25^ Additionally, in a study by Cristel et al, the intravenous administration of TXA during surgery did not yield a decreased incidence of ecchymosis following rhinoplasty procedures.^26^

TXA acts as an antifibrinolytic by competitively inhibiting plasminogen binding to fibrin, thereby stabilizing clots and reducing fibrinolysis. It prevents clot breakdown rather than promoting clot formation. The observed discrepancy between our results and those from previous studies indicates that the local infiltration route may not confer the same advantages in rhinoplasty as oral or intravenous administration. This variation in effectiveness may be attributed to several factors, including differences in drug distribution, absorption, or the distinctive vascular anatomy of the nasal region when compared with other areas of the face. For instance, Vural et al reported that the application of TXA-soaked pledgets to the nasal osteotomy sites resulted in diminished eyelid edema on postoperative Day 1, along with reduced periorbital ecchymosis.^27^ Similarly, Sezen Göktaş et al conducted a 4-arm RCT that demonstrated that the topical application of TXA yielded the lowest postoperative edema scores at all measured time points, as well as a significant reduction in ecchymosis in comparison to intravenous or oral TXA.^28^ Until the role of local TXA is more precisely defined, surgeons may need to continue relying on established techniques for the mitigation of periorbital edema and ecchymosis, such as controlled hypotension, head elevation, and cooling methods to minimize postoperative complications such as ecchymosis and edema.

It is important to note that our results are specific to the protocol employed in this study, which included a standardized Joseph open septorhinoplasty technique, subperiosteal dissection before osteotomy, and controlled anesthesia with strict blood pressure monitoring. Within this controlled environment, local infiltration of TXA conferred no additional benefit in reducing ecchymosis, edema, or bleeding. These findings underscore that local infiltration should not be assumed to have the same efficacy as systemic administration, which has been shown in previous studies to reduce intraoperative blood loss and postoperative bruising.^29^ Although our data are protocol specific, they provide valuable negative evidence and suggest that further research comparing local and systemic routes directly is warranted. These discrepancies may be explained by differences in TXA dosage (weight-based intravenous or high-dose local vs our low-concentration infiltration), variations in timing of administration, and differences in tissue distribution between facelift and rhinoplasty procedures.

Our study presents certain limitations, primarily associated with sample size. Although we included 80 patients, who were evenly distributed across the groups, the *P* values, which approached but did not attain significance, indicate that a larger sample size may have produced different results. This limitation is particularly significant when evaluating ecchymosis 1 week postsurgery, where the values were near the significance threshold. The study's prospective design, while facilitating careful control of variables, constrained our recruitment capacity. Furthermore, despite our efforts at standardization, the subjective nature of edema and ecchymosis assessments may have resulted in measurement variability.

Nonetheless, our study adopted a comprehensive triple-blind design to minimize bias, with a strong focus on proper blinding and concealment methods. Data collection was performed systematically, adhering to schedules and ensuring patient compliance with photographic submissions. An experienced and impartial analyst conducted the analysis to minimize the risk of interpretation errors. To our knowledge, this is among the first triple-blind RCTs examining low-dose local TXA infiltration in rhinoplasty. Although Vaghardoost et al previously studied higher-dose local TXA, our study differs in methodology and concentration, offering complementary evidence.

Another point worth noting is that patients with severe septal deviation or significant bony vault deformity were excluded from this trial. This selection criterion aimed to minimize variability, as such patients often require more extensive osteotomies and cartilage grafting, which could independently affect postoperative edema and ecchymosis. By limiting our sample to cases suitable for the Joseph open septorhinoplasty approach with standard low-to-low osteotomy, we ensured consistency in the surgical technique. Although this improves internal validity, it may restrict the applicability of our findings to patients with more complex nasal deformities.

We did not include an intravenous TXA arm or a split-face design, both of which might have provided additional comparative data and strengthened our conclusions. Studies directly comparing different administration routes within the same patient population would yield valuable comparative data.

Additional potential sources of bias include the lack of standardized patient photographs, reliance on a single blinded assessor for image evaluation, and the absence of strict control over patient-related factors such as head elevation, use of cold compresses, and postoperative activity, all of which may have influenced the outcomes.

A significant limitation was the exclusion of patients with pre-existing hypotension, which may have introduced selection bias and constrained the generalizability of the results. Implementing alternative exclusion criteria or stratified inclusion might yield different outcomes. Moreover, this study was conducted by a single surgeon, ensuring consistency in technique but potentially limiting external applicability. Future multicenter trials with larger sample sizes and more diverse patient populations are recommended to validate and extend these findings.

## CONCLUSIONS

Our randomized, triple-blind study found that local infiltration of TXA did not significantly reduce periorbital edema, ecchymosis, or intraoperative bleeding in rhinoplasty patients compared with the placebo. These findings contrast with previous studies of oral and intravenous TXA, underscoring that local infiltration may not replicate the benefits of systemic administration in rhinoplasty. Further investigations involving larger sample sizes are imperative to conclusively ascertain the efficacy of local TXA infiltration in mitigating postrhinoplasty complications.

## Supplemental Material

This article contains supplemental material located online at https://doi.org/10.1093/asjof/ojaf172.

## Supplementary Material

ojaf172_Supplementary_Data
