# Supplementary material for: The Impact of Local Tranexamic Acid Infiltration on Periorbital Ecchymosis and Edema in Rhinoplasty: A Triple-Blinded Randomized Controlled Trial
Source: Aesthet Surg J Open Forum. 2025 Dec 23;8:ojaf172. doi: 10.1093/asjof/ojaf172 (PMC12854225; doi:10.1093/asjof/ojaf172)
Supplement: ojaf172_Supplementary_Data [file ojaf172_supplementary_data.docx]

# Supplementary Table S1. Grading Scales for Periorbital Oedema and Ecchymosis (Mehdizadeh et al.)

## Oedema Scale

| Grade | Description |
| --- | --- |
| 0 | No oedema |
| 1 | Mild oedema limited to the medial canthus |
| 2 | Oedema involving less than half of the eyelid |
| 3 | Oedema involving more than half of the eyelid |
| 4 | Severe oedema with eyes completely closed |

## Ecchymosis Scale

| Grade | Description |
| --- | --- |
| 0 | No ecchymosis |
| 1 | Ecchymosis covering less than one third of eyelid surface area |
| 2 | Ecchymosis covering one third to two thirds of eyelid surface area |
| 3 | Ecchymosis covering more than two thirds of eyelid surface area |
| 4 | Ecchymosis covering entire eyelid and surrounding periorbital region |
